# Experimental Realization of Tunable Metamaterial Hyper-transmitter

**DOI:** 10.1038/srep33416

**Published:** 2016-09-15

**Authors:** Young Joon Yoo, Changhyun Yi, Ji Sub Hwang, Young Ju Kim, Sang Yoon Park, Ki Won Kim, Joo Yull Rhee, YoungPak Lee

**Affiliations:** 1 Department of Physics and RINS, Hanyang University, Seoul, South Korea; 2 Department of Physics, Sungkyunkwan University, Suwon, South Korea; 3 Advanced Institutes of Convergence Technology, Seoul National University, Suwon, South Korea; 4 Department of Display Information, Sunmoon University, Asan, South Korea

## Abstract

We realized the tunable metamaterial hyper-transmitter in the microwave range utilizing simple planar meta-structure. The single-layer metamaterial hyper-transmitter shows that the transmission peak occurs at 14 GHz. In case of the dual-layer one, it is possible to control the transmission peak from 5 to 10 GHz. Moreover, all the transmission peaks reveal transmission over 100%. We experimentally and theoretically investigated these phenomena through 3-dimensional simulation and measurement. The reason for being over 100% is also elucidated. The suggested hyper-transmitter can be used, for example, in enhancing the operating distance of the electromagnetic wave in Wi-Fi, military radar, wireless power transfer and self-driving car.

In the present days, mankind lives in a number of waves, such as sound waves and electromagnetic (EM) waves including light. In particular, the EM waves play great roles to enrich the human life. People can see the objects, use the cell phones, and send and receive the information using the EM waves. In addition, mankind often absorbs or focuses the EM waves at specific positions, and transfer them far way according to the human requirements.

Metamaterials (MMs), defined as materials which are not existing in nature, are artificial materials providing unobservable characteristics in nature, such as negative refraction, perfect absorption and perfect transmission, based on the periodic arrangement of meta-atoms made in a size smaller than the wavelength of incident EM wave^1^,^2^,^3^,^4^. MMs can be applied to various fields such as optical cloaking, perfect lenses and perfect absorbers, because of the special properties originating from the negative-index media^5^,^6^,^7^,^8^. One of the most dramatic properties of negative-refractive MMs is that MM can be a perfect lens, which was theoretically reported by Pendry^9^. The perfect lens can make the spread of light be focused at specific point without any deterioration. Since the Pendry’s work, many researchers have attempted to realize the experimental superlens^10^,^11^,^12^,^13^,^14^,^15^. The main key needed for superlens is its ability to enhance the evanescent waves, resulting in sharper image and overcoming the near-field limitation. In order to release the near-field restraint, especially, far-field superlens and hyper-lens have been studied in the optical range^16^,^17^,^18^,^19^,^20^,^21^,^22^. The difference between the near-field and the far-field superlens is the diffraction limit. In case of the near-field, the diffraction limitation is less than λ, the operating wavelength. Based on previous reports, the diffraction limit for the near-field superlens is from λ/20 to λ/5^23^,^24^,^25^,^26^. For the far-field superlens, on the other hand, the focal length is over λ, which means that high-resolution images can be reconstructed by collecting the far-field signals^27^. The far-field superlens was proposed to project a sub-diffraction-resolution image into the far field^16^. In addition, the far-field superlens not only improves the evanescent waves but also converts them into propagating waves.

This research intends to introduce new type of planar MM hyper-transmitter, which is a kind of far-field superlens or hyper-lens. The MM hyper-transmitter shows that the transmission peak over 100% can be controlled in the 5–10 GHz range. In addition, it is also possible to transmit far away the EM wave with respect to the general antenna system. The MM hyper-transmitter can be used for transmitting the EM wave for long distance in such as Wi-Fi, military radar, wireless power transfer and self-driving car.

To send the EM-wave signal, we used two antennas; one is a transmitting antenna and the other is a receiving one. As shown in Fig. 1a, the distance between transmitting and receiving antennas should be 6 × λ or some larger, where λ is the working wavelength, by considering the wavelength of EM wave. In the case of λ = 10 cm (3 GHz), the distance between transmitting and receiving antennas can be 1 m. The emitted EM waves spread and go out from the transmitting antenna to the receiving one. The spread of EM-wave radiation is actually close to 20 degrees. When the EM wave approaches the receiving antenna, the maximum magnitude of transmission should be 100%. On the other hand, if we utilize the hyper-transmitter (including superlens and hyper-lens) based on MM, the spreading EM wave can be focused on the receiving antenna (Fig. 1b). In this case, the transmission magnitude can be greater than 100%. We suggest MM hyper-transmitter, based on this flow of ideas.

Figure 2a shows the designed single-layer MM for hyper-transmitter. The meta-atom consists of two patterned metallic layers, separated by a dielectric substrate. The dielectric substrate is FR-4 with a thickness of 0.8 mm, and a dielectric constant of ε = 4.3 and a dielectric loss tangent of 0.025. The patterned metal is copper with an electric conductivity of σ = 5.8 × 10^7^ S/m, and the thickness of metallic layer is *t*_*c*_ = 36 μm. The metallic pattern is a ring structure, and the optimum value for radius is 7 mm, with a width of 3.5 mm and a periodicity of 18.2 mm. The patterned metallic layers at the front and the back are aligned. The incident EM wave is polarized that the electric and the magnetic fields are parallel to *y* and *x* axes, respectively (Fig. 2a). The propagation of EM wave is *z* direction in the environment of free space.

The simulated and the experimental transmission spectra of suggested structure are illustrated in Fig. 2b for a wide range of 4–16 GHz. The experimental transmission spectrum is in good agreement with the simulated one. The transmission spectra reveal transmission of 105% at 14.52 GHz and of 104% at 14.69 GHz for simulation and experiment, respectively. A noteworthy aspect in Fig. 2b is that the experimental transmission magnitude is greater than 100%. This experimental phenomenon looks very interesting. As aforementioned for Fig. 1, if the spreading EM wave is focused on the receiving antenna by the suggested structure, the transmission magnitude can be greater than 100%. The difference between simulated and experimental transmission is considered by that in simulation the transmission is drastically changed according to the position of receiving antenna. To confirm the origin of high transmissions at 4.24 and at 14.52 GHz, we investigated the surface currents, the induced electric field and the induced magnetic field (see Figs S1 and S2 in Supplementary). The transmission magnitude at 4.24 GHz is high. The difference between transmission at 4.24 and 14.52 GHz is the resonance mode. The resonance mode at 4.24 GHz is the magnetic resonance. On the other hand, the resonance at 14.52 GHz is the electric resonance. In the simulation, the resonance transmission peak at 14.52 GHz turns out to be induced by the parallel surface currents on the metallic layers. Therefore, the transmission is enhanced because the electric field in the sample is formed in the same direction as the electric field direction of the incident EM wave. The refractive index at 14.52 GHz is calculated to be negative (refer to Section II in Supplementary). To enhance the understanding, we also simulated the 3-dimensional EM-wave distribution around the single-layer MM (shown in Fig. 2c). It can be confirmed that the focusing point is around 30λ (~60 cm from the single-layer MM). This is similar to the previously-reported far-field superlens^22^. In general, the standard focusing is that the object is converted to the image by using the conventional lens or superlens. In case of the suggested single-layer MM, on the other hand, the main effect is that the EM wave can be sent further away.

To confirm the focusing of EM wave, we measured 3-dimensional EM-wave patterns in free space, single layer without pattern and single-layer MM according to the angle of receiving antenna (Fig. 3a). For the free-space case, the transmission according to the angle is found to be in a Gaussian shape with the center at zero degree. In case of the single layer without pattern, the transmission appears to be lower than that for the free-space case because of the dielectric loss in FR-4 substrate. For the single-layer MM, on the other hand, the transmission turns out to be higher than that for the free-space case only from zero to 5 degrees, but is shown to be lower over 5 degrees. In other words, EM wave can be focused by the single-layer MM. To verify the EM wave according to the distance from sample, we measured comparatively EM-wave magnitudes for the free-space case and single-layer MM. In free space, as shown in Fig. 3b, the transmission decreases in proportion to the square of distance. Meanwhile, the transmission shows a point of inflection for the single-layer MM. This result might indicate that EM wave is focused at certain point by the single-layer MM.

In order to expand the application areas, we designed dual-layer MM which can control the transmission peaks. As shown in Fig. 4a, the dual layers of MM is made of four separated copper layers, both of which are deposited on a dielectric FR-4 board. The distance *d* is a gap between the two sets of layers. To understand the effects of distance *d*, we simulated the transmission spectra of 4–16 GHz according to *d* from 8 to 40 mm. Figure 4b shows the simulated transmission spectra according to the distance. The transmission spectrum for *d* = 8 mm presents transmission of 51.5% at 13.88 GHz. It is easily found that this transmission peak position is shifted to lower frequency with increasing the distance. Another transmission peak occurs for *d* = 16 mm. This transmission peak is also shifted to lower frequency by increasing the distance. The transmission frequencies can be adjusted by controlling the distance between the two layers. On the other hand, the first transmission peak disappears at *d* = 12 mm, and the second transmission peak also disappears at *d* = 24 mm, which will be discussed later. Another noteworthy phenomenon in Fig. 4b is that the transmission is over 100% for the distance from 20 to 40 mm. In the simulated transmission spectra, the transmission of the first peak turns out to be 124% at 8.31 GHz, 133% at 7.18 GHz, 133% at 6.34 GHz, 131% at 5.72 GHz, 131% at 5.23 GHz, and 155% at 4.92 GHz with *d* = 20, 24, 28, 32, 36 and 40 mm, respectively. For the dual-layer MM, the transmission frequency can be obtained by


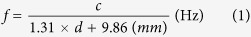


Here, *c* is the velocity of light. According to this calculation, the wavelength of transmission frequency turns out to increase linearly with respect to distance *d*. The transmission for dual-layer MM is enhanced by the electric resonance, which is caused by the parallel surface currents on both front and back metallic layers. The distance between two layers manipulates the transmission frequency. In other words, the focused transmission frequency can be controlled by the distance between two layers.

To confirm the results of simulation, we have measured the transmission spectra in a range of 4–18 GHz. The experimental transmission spectra are similar to the simulation ones (Fig. 5a–c). For *d* = 12 mm, as aforementioned, there is no transmission peak in both simulation and experiment (Fig. 5a). In case of *d* = 20 mm (Fig. 5b), high-transmission peak emerges, with transmission of 124% at 8.34 GHz and of 133% at 8.51 GHz for simulation and experiment, respectively. In addition, the second transmission peak appears around 13.64 GHz. For *d* = 30 mm (Fig. 5c), two transmission peaks are evident. The low-frequency peak reveals transmission over 100% for the experiment: a transmission of 152% at 6.04 GHz. The middle-range peak also shows certain transmission: transmission of 30% at 10.2 GHz and of 37% at 10.37 GHz for simulation and experiment, respectively.

We examined the trend for the transmission peaks in both simulation and experiment (Fig. 5d). The transmission peaks are shifted to lower frequency according to distance *d*. Moreover, the experimental transmission-peak positions are in good agreement with those of the simulation. As mentioned above, there is no peak for *d* = 12 and 24 mm due to the fact that the wavelength of expected peak position is equal to the distance between two layers. We investigated the transmission magnitudes of the first and the second peaks according to the distance (Fig. 5e,f, respectively). For the first transmission peak, the trend of the experimental transmission magnitudes is similar to the simulation one. The noteworthy aspect in Fig. 5e is that the magnitudes of the simulated and the experimental transmission for *d*  =  20 to 40 mm are over 100%. At the same time, the trend of the experimental transmission magnitudes for the second peak is in good agreement with the simulation (Fig. 5f).

To understand the focused EM wave, we also simulated the 3-dimensional EM-wave distribution around the hyper-transmitter and measured the transmission pattern (Fig. 6). Figure 6a,b show EM-wave distribution and the transmission pattern for *d* = 20 and 30 mm, respectively. First of all, the EM-wave distribution without sample reveals that EM waves spread and go out from the transmitting antenna for both *d* = 20 and 30 mm. In case of the existence of sample, on the other hand, the EM waves are focused after passing through the hyper-transmitter. Based on these results, it is found that EM wave can be sent further away using the hyper-transmitter.

To further compare the results of simulation with those of experiment, we measured the transmission pattern according to the angle and the position of the receiver antenna (bottom of Fig. 6a,b). Positions ①, ② and ③ indicate 30, 40 and 50 cm away from the sample, respectively. For the distance of 20 mm, the transmission pattern without sample shows a Gaussian shape with the center at zero degree, which are same at all the distances. In case of the hyper-transmitter, however, the transmission-peak pattern was significantly varied by the position. At position ①, although the transmission is lower than 100% at zero degree, but it is found easily that the transmission is greater than 100% at 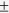
4 degrees. This phenomenon is also similar to position ②. On the other hand, the transmission at position ③ comes to be very high (126%) in a wide range of angle around zero. The trend of transmission pattern for *d* = 30 mm differs from the distance of 20 mm. The transmission is higher than that in free space from zero to 7 degrees for position ①, but is shown to be lower over 7 degrees. For position ②, the transmission pattern is the most narrow and the transmission is higher than that at the other positions. On the other hand, the transmission pattern is slightly broadened for position ③. In Fig. 6, the distribution of transmission looks high from the top and the bottom of sample due to the diffraction phenomenon in the simulation. Based on these results, it is thought that the frequency modulation is possible by the distance between two layers, as well as the focal point of EM wave is also changed (Fig. 5). In order to obtain exactly the focal point, we also measured the magnitude of transmission peak by the position of receiving antenna. In addition, we calculated the experimental focal point though the calculation of normalized transmission. From the results, the focal point for *d* = 20 mm turns out to be nearly 115 cm from the sample. In case of *d* = 30 mm, on the other hand, the focal point is about 100 cm from the sample (refer to Section III in Supplementary). In general, the perfect lens is possible only when the refractive index is −1^9^. Especially, the perfect lens can collect the spread light at one point. On the other hand, our suggested MMs can send EM wave further away, although it is lossy because the refractive index is not −1.

It is experimentally confirmed that the frequency-tunable hyper-transmitter for 5–10 GHz can be manufactured by simple MM patterning and simple method, and the reasons are elucidated in detail. It is also found that the surface current flows well on the MM pattern, leading to high transmission peak. For the dual-layer hyper-transmitter, the transmission frequency can be even tuned simply by controlling the distance between two layers, even though the single-layer one is also capable to focus EM waves. In addition, the focal points were exactly confirmed. The dual-layer hyper-transmitter can be applied to the long-distance transfer. The results suggest possibility of active MM which can apply for transmitting EM waves for long distance, in such as Wi-Fi, military radar and self-driving car.

## Method Summary

### Electromagnetic-wave transmission measurement

The transmission spectra were measured in a microwave anechoic chamber using a Hewlett-Packard E8363B network analyzer connected to linearly-polarized microwave standard-gain horn antennas and calibrated in free space. Two horn antennas, which were 300 mm wide and 200 mm long, were used: one for transmitting the microwave beam on the sample and the other for receiving the transmitted beam (distance between two horn antennas: 1.0 m). The transmission was obtained by *T*(*ω*) = |*S*_*21*_|^2^. |*S*_*21*_|, which are the scattering parameters for transmission.

### Electromagnetic-wave simulation

**Figure 2:**
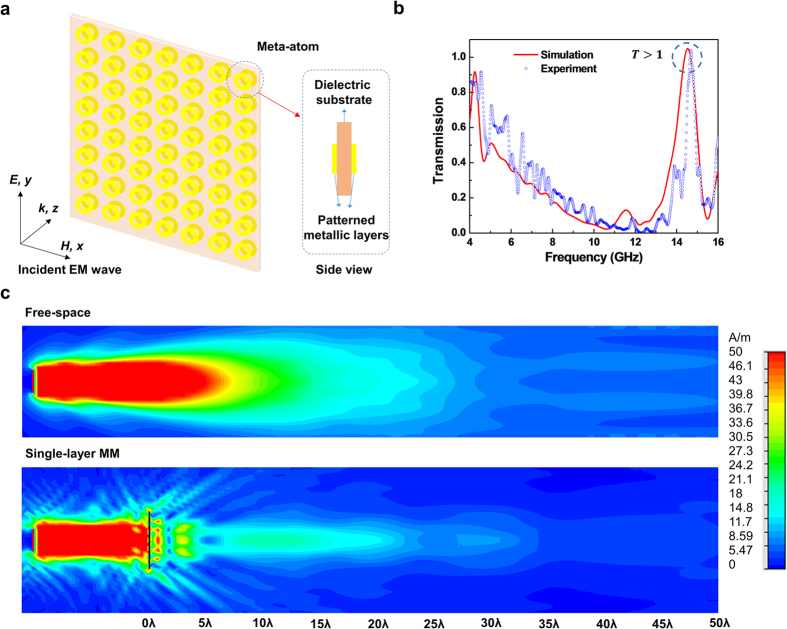
Single-layer MM, and results of EM-wave transmission spectrum in 4–16 GHz. (**a**) Mimetic diagram of designed MM and meta-atom. In simulation, the incident EM wave of electric field (E) parallel to *y* axis, and magnetic field (H), parallel to *x* axis propagates in the direction of *k* vector, parallel to *z* axis. (**b**) Transmission spectrum for single-layer MM hyper-transmitter. Transmission around 14.6 GHz is higher than 100% in both the simulation and the experiment. (**c**) Simulation on 3-dimensional EM wave pattern for single-layer MM. EM waves passing through the sample are gathered around 30λ.

**Figure 5:**
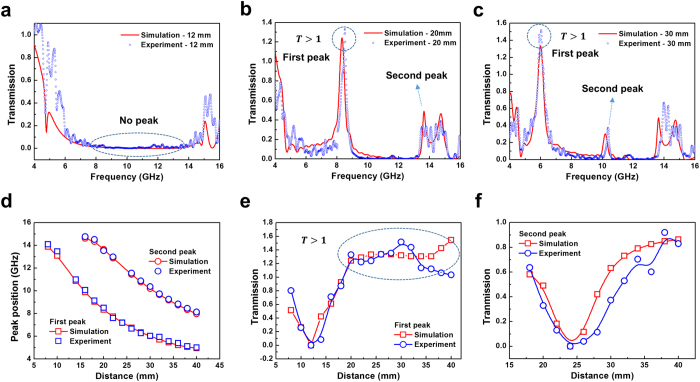
Transmission of two-layer MM hyper-transmitter. (**a**) Simulated and experimental transmission spectra for *d* = 12 mm. For *d* = 12 mm, as aforementioned, there is no transmission peak in both simulation and experiment. (**b**) Simulated and experimental transmission spectra for *d*  =  20 mm. High-transmission peak is found, with transmission of 124% at 8.34 GHz and of 133% at 8.51 GHz for simulation and experiment, respectively. (**c**) Simulated and experimental transmission spectra for *d* = 30 mm. The low-frequency peak shows transmission over 100% for both experiment and simulation, with transmission of 152% at 6.04 GHz and 133% at 6 GHz, respectively. (**d**) Change of the transmission peak position according to the distance between two layers. When the distance is increased, the transmission peaks are shifted to lower frequency. (**e**) Change of the magnitude of first transmission peak according to the distance between two layers. The experimental transmission magnitude for the first peak is in good agreement with the simulation. Moreover, the transmission is over 100% when the distance is higher than 20 mm. (**f**) Change of the magnitude of second transmission peak according to the distance between two layers. The experimental magnitude for the second peak is similar to the simulation.

**Figure 6:**
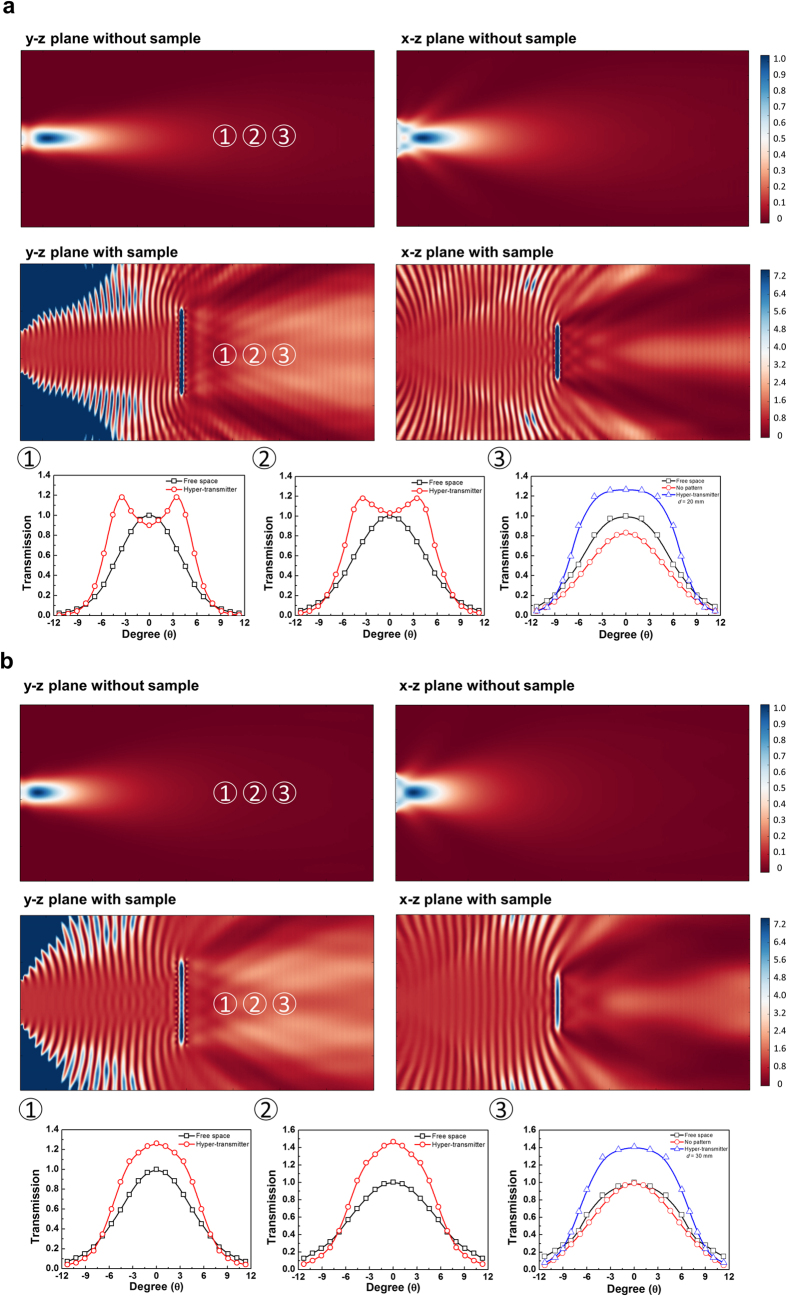
Simulation on the 3-dimensional EM-wave distribution and experimental results of the transmission peak magnitude by the angle and the position of receiving antenna. (**a**) Simulation on 3-dimensional EM wave pattern and experimental results of the transmission peak magnitude for *d* = 20 mm by the position of receiving antenna. (**b**) Those for *d* = 30 mm.

The simulations in Figs 2b, 5 and 6, and Supplementary Figs S1, S2 and S3 were performed using CST Microwave Studio^®^ 2011. The simulations were performed using a transient solver.

## Additional Information

**How to cite this article**: Yoo, Y. J. *et al*. Experimental Realization of Tunable Metamaterial Hyper-transmitter. *Sci. Rep.* **6**, 33416; doi: 10.1038/srep33416 (2016).

## Supplementary Material

Supplementary Information

## Figures and Tables

**Figure 1:**
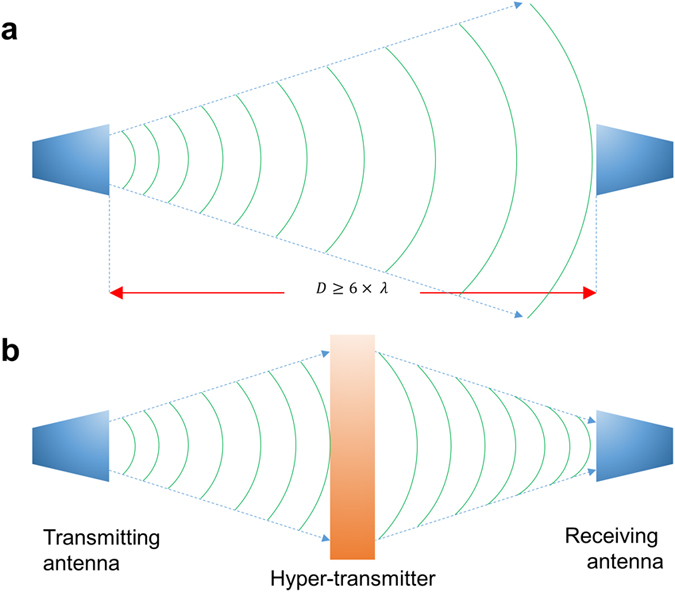
Mimetic diagram of MM hyper-transmitter. (**a**) To send EM-wave signal, we used two antennas: one is a transmitting antenna and the other is a receiving one. The emitted EM waves spread and go out from the transmitting antenna to the receiving one. (**b)** If we utilize the hyper-transmitter based on MM, the spread of EM wave can be focused on the receiving antenna.

**Figure 3:**
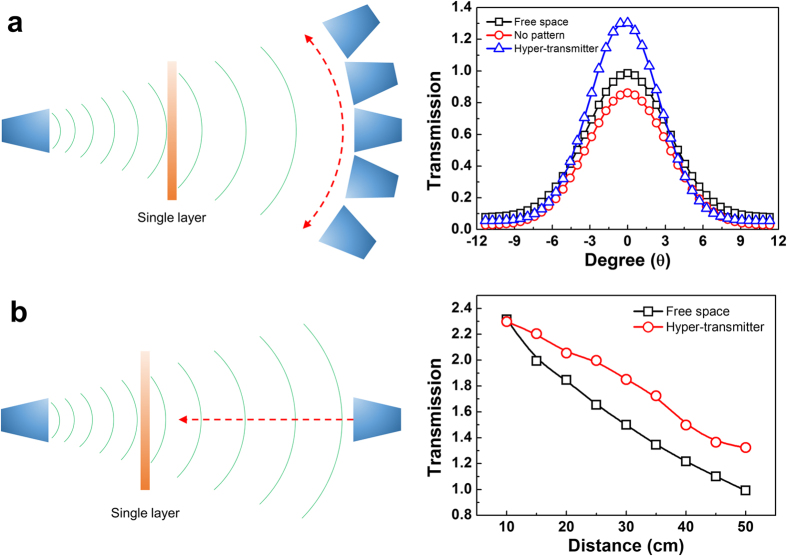
Change of EM-wave transmission by the angle and the position of receiving antenna. (**a**) Schematic of transmission measurement with respect to the angle of receiving antenna and change of the transmission peak according to the angle of receiving antenna. The transmission in free space according to the angle is found to be in a Gaussian shape with the center at zero degree. The transmission for only the substrate without pattern appears to be lower than that for the free-space case. On the other hand, the magnitude of transmission peak for the single-layer hyper-transmitter comes to be higher than that for the free-space case from zero to 5 degrees, but is changed to be lower over 5 degrees. (**b)** Schematic of transmission measurement with respect to the position of receiving antenna and change of the transmission peak according to the position of receiving antenna. The transmission in free space decreases in proportion to the square of distance. The transmission magnitude for the single-layer MM hyper-transmitter is higher than that for the free-space case, even though it has a point of inflection at the beginning.

**Figure 4:**
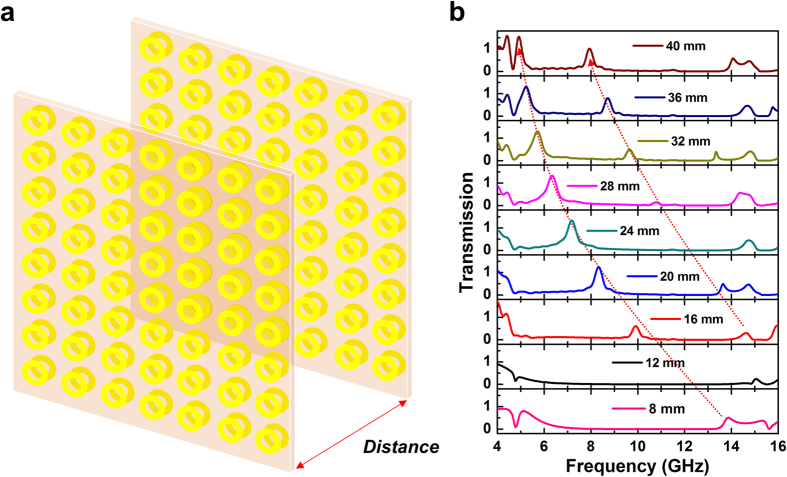
Change of EM-wave transmission according to the distance between two layers. (**a**) Schematic of controlling the distance between two layers. (**b**) Change of the simulated transmission spectrum according to the distance between two layers by 9 steps (8, 12, 16, 20, 24, 28, 32, 36 and 40 mm). As the distance between two layers is increased, the transmission peak is shifted to lower frequency. In addition, when the distance is over 16 mm, new transmission peak appears between existing peaks.
