# Supplementary material for: Experimental Realization of Tunable Metamaterial Hyper-transmitter
Source: Sci Rep. 2016 Sep 15;6:33416. doi: 10.1038/srep33416 (PMC5024307; doi:10.1038/srep33416)
Supplement: Supplementary Information [file srep33416-s1.doc]

**Supplementary Information for ‘Experimental Realization of Tunable Metamaterial Hyper-transmitter’**

Young Joon Yoo, Changhyun Yi, Ji Sub Hwang, Young Ju Kim, Sang Yoon Park, Ki Won Kim, Joo Yull Rhee, YoungPak Lee

Here, in this Supplementary Information, we present details on the mechanism of transmission, which is induced by the electric resonance and on the calculation of refractive index for the transmission peak, and changes of the transmission peak magnitude according to the angle and the position of the receiving antenna.

**I. Distribution of the surface current on the front and the back metallic layers.**

To demystify the mechanism of transmission, we investigated the surface currents, the induced electric field and the induced magnetic field at 4.24 and 14.53 GHz. Figure S1 shows the distribution of surface current on the front and the back metallic layers, and the induced fields in the sample at 4.24 GHz. When the incident electromagnetic (EM) wave with phase = 0 meets the metamaterial (MM), for the transmission at 4.24 GHz, the anti-parallel currents on the front and the back plates are exhibited. On the other hand, the relatively-strong surface current at the outer edge of ring flows from top to bottom for the transmission peak at 14.53 GHz. The surface currents at the front and the back metallic layers induce the electric field in the substrate.


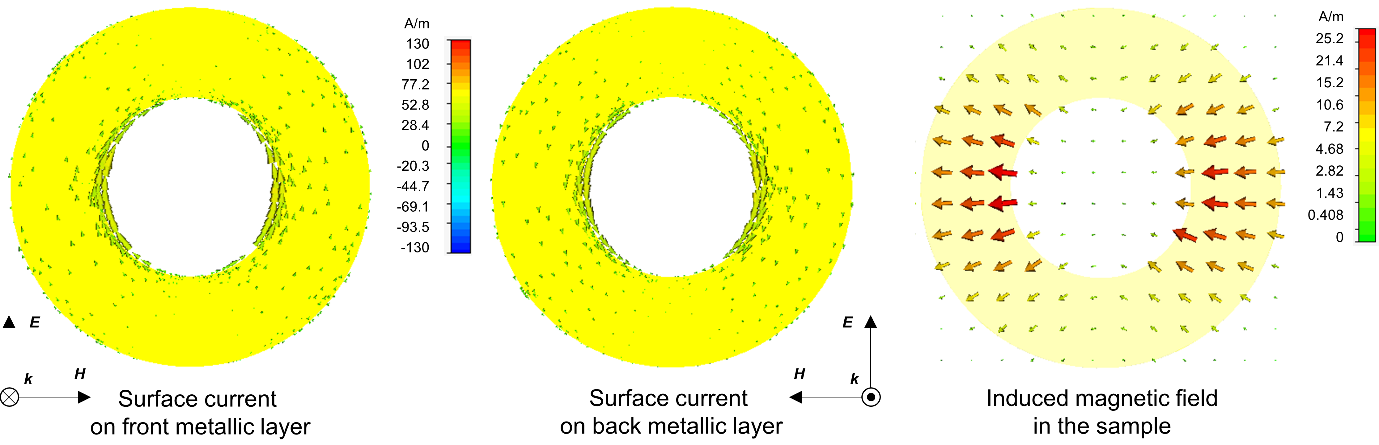


**Figure S1 | Distribution of the surface current and the induced magnetic field at 4.24 GHz.** When the incident EM wave with zero phase meets the single-layer hyper-transmitter, for the transmission at 4.24 GHz, the anti-parallel surface currents flow from bottom to top plane at the front and back metallic layers. This phenomenon is induced by the magnetic field in the opposite direction as the incident magnetic field.


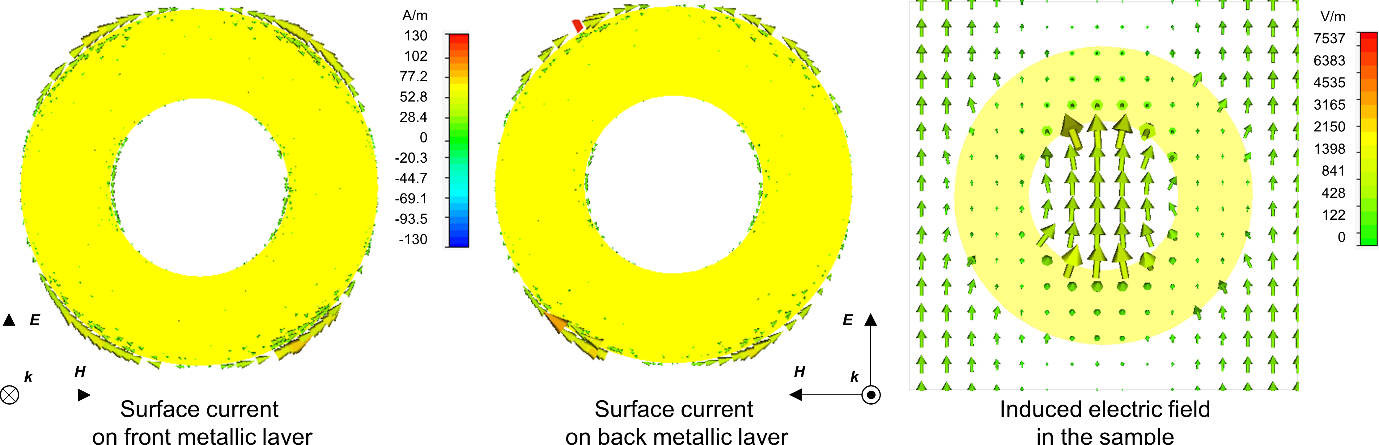


**Figure S2 | Distribution of the surface current and the induced electric field at 14.53 GHz.** For the transmission peak at 14.53 GHz, when the incident EM wave with zero phase meets the single-layer hyper-transmitter, the surface currents flow from bottom to top plane at the front and back metallic layers. This phenomenon is induced by the electric field in the same direction as the incident electric field.

**II. Calculation for the refractive index around 14.35 GHz.**

To understand the high transmission (over 100%) around 14.35 GHz, the refractive index is calculated by using the improved version of the standard retrieval method. The refractive index can be derived by the following expression^1^;


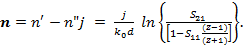
 (1)

Here, *z, S*_11_ and *S*_21_ are the impedance, the scattering parameters of reflection and transmission, respectively. Figure S3 presents the transmission, the S-parameter phase and the refractive index for single-layer hyper-transmitter. The S-parameter phase change rapidly from the inflection point of transmission, and the change in S-parameter is related to the refractive index. As shown in bottom of Fig. S3, the real part of refractive index is rapidly changed around 14.54 GHz. Moreover, the real part reveals a negative index.


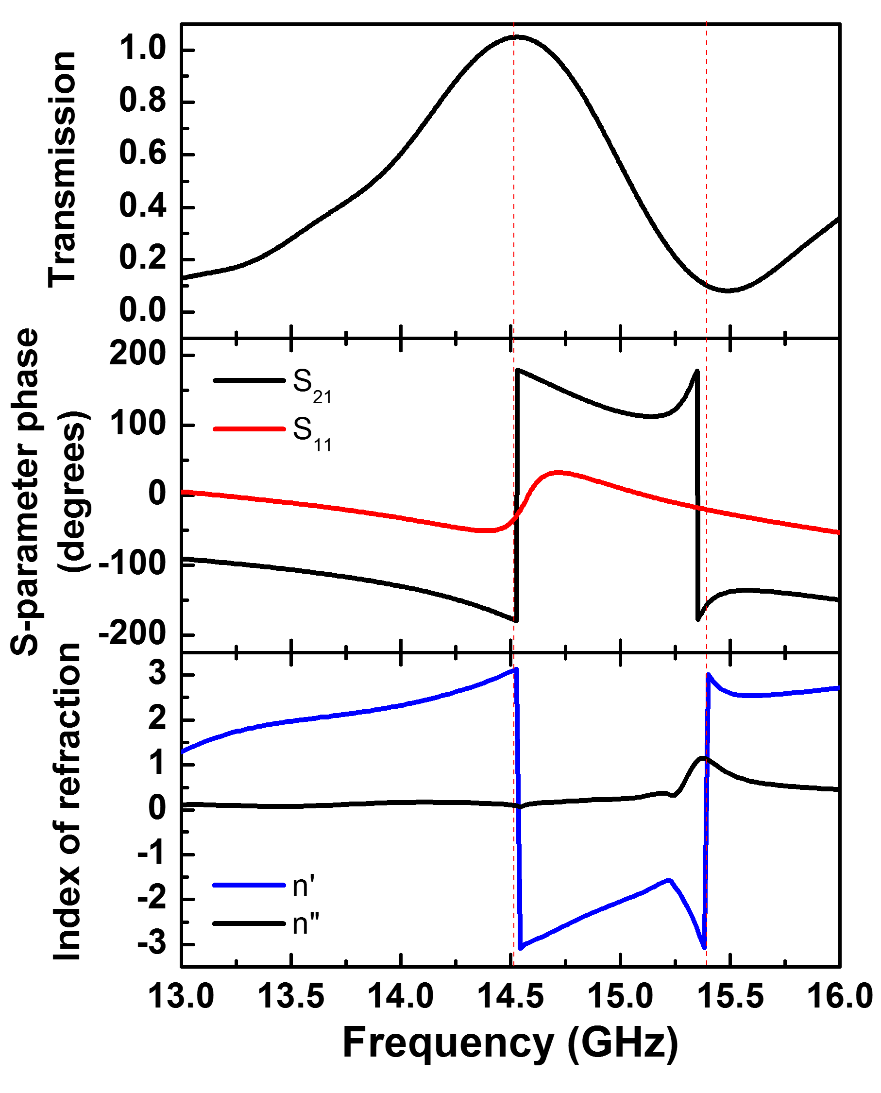


**Figure S3 | Results of the transmission, S-parameter phase and the refractive index for single-layer hyper-transmitter.** The S-parameter phase changes rapidly from the inflection point of transmission, and the change in S-parameter is related to the refractive index. The real part of refractive index is negative around the transmission peak.

**III. Changes of the transmission peak magnitude according to the angle and the position of receiving antenna.**

In order to confirm the behavior of EM wave according to the position from sample, we also measured the EM-wave distribution in the free-space case and for dual-layer hyper-transmitter at *d* = 20 and 30 mm [shown in Fig. S4]. When the position is 50 cm in free space, we assumed the transmission of 100%. In free space, the transmission decreases in proportion to the square of position for both cases as shown in Figs. S4a and S4b. The magnitude of transmission peak for *d* = 20 mm shows that the relative transmission is higher than that in free space when the position of receiving antenna with respect to hyper-transmitter is 35 cm. For the *d* = 30 mm, on the other hand, when the position of receiving antenna with respect to hyper-transmitter is around 20 cm, the transmission is significantly increased with respect to that in free space. These results also indicate that the EM waves are focused nicely and controllably at certain point by the dual-layer hyper-transmitter. In order to obtain exactly the focal point, in addition, we calculated the experimental focal point though the calculation of normalized transmission. Figures S4c and S4d present the normalized transmission for dual-layer hyper-transmitter at *d* = 20 and 30 mm, respectively. Based on the obtained results, for *d* = 20 mm, the focal point is nearly 115 cm. The relative transmission at focal position is close to 170%. On the other hand, the focal point for *d* = 30 mm turns out to be about 100 cm. In this case, the relative transmission turns out to be nearly 194%. From these results, the dual-layer hyper-transmitter can be efficiently applied to the long-distance transfer.


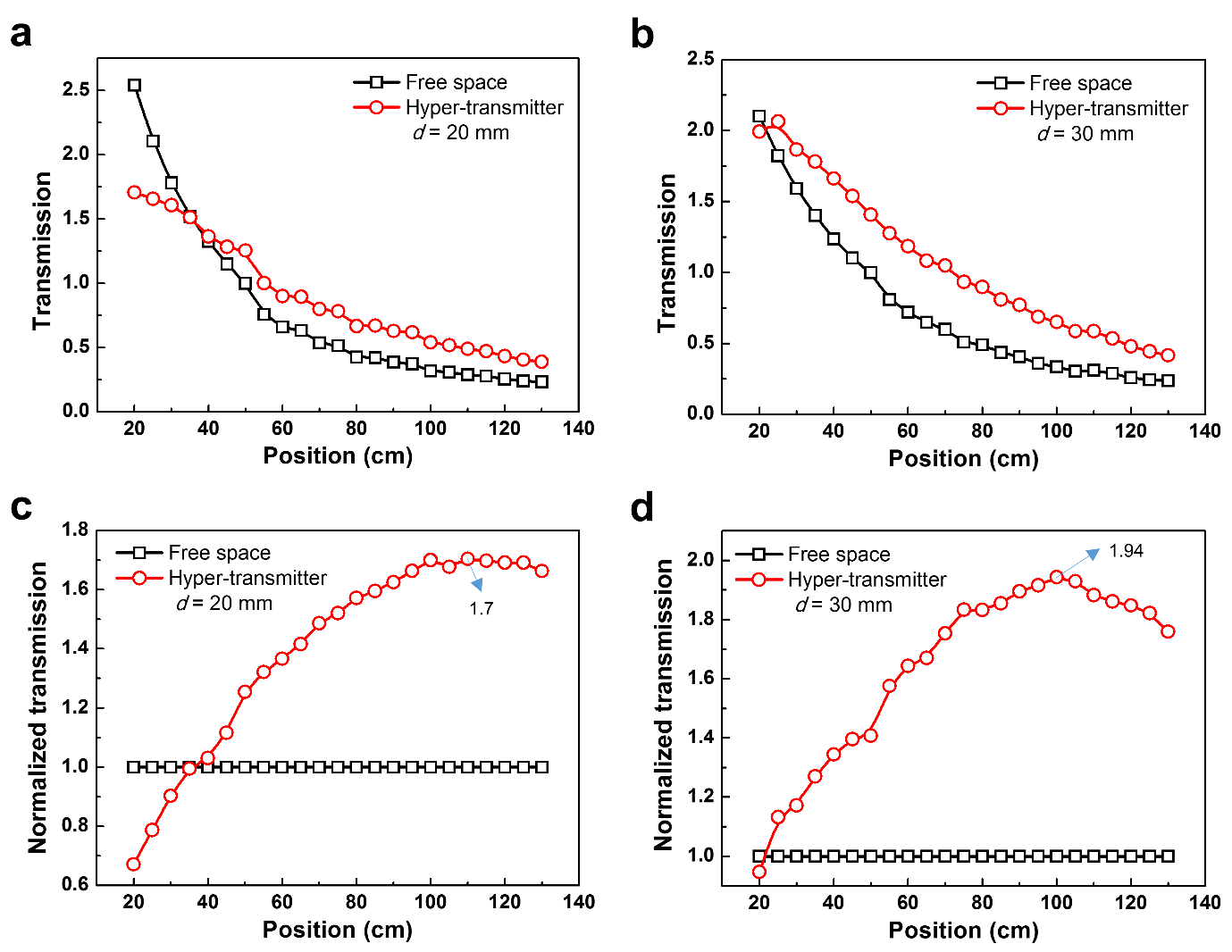


**Figure S4 | Experimental results of transmission peak magnitude by the position of receiving antenna. a,** Experimental results of the transmission for *d* = 20 mm by the position of receiving antenna. The transmission magnitude in free space is increased in proportion to the square of position. On the other hand, the magnitude of transmission peak shows that the relative transmission is higher than that in free space when the position of receiving antenna with respect to hyper-transmitter is 35 cm. **b,** Experimental results of the transmission for *d* = 30 mm by the position of receiving antenna. When the position of receiving antenna with respect to hyper-transmitter is around 20 cm, the transmission through hyper-transmitter is significantly increased with respect to that in free space. **c,** Normalized transmission for *d* = 20 mm according to the position of receiving antenna. **d,** Normalized transmission for *d* = 30 mm according to the position of receiving antenna.

**Reference**

1. Smith, D. R., Vier, D. C., Koschny, T., Soukoulis, C. M., *Phys. Rev. E* **71**, 036617 (2005).
